# Prehospital and Emergency Care Perspectives to Define Pediatric Critical Illness and Injury

**DOI:** 10.5811/westjem.48526

**Published:** 2026-01-09

**Authors:** Sriram Ramgopal, Rebecca E. Cash, Christian Martin-Gill, Ashley Hayes, Leonard Barrera, Christopher M. Horvat, Michelle L. Macy

**Affiliations:** * Northwestern University Feinberg School of Medicine, Ann & Robert H. Lurie Children’s Hospital of Chicago, Division of Emergency Medicine, Chicago, Illinois; † Harvard Medical School, Massachusetts General Hospital, Department of Emergency Medicine, Boston, Massachusetts; ‡ University of Pittsburgh School of Medicine, Department of Emergency Medicine, Pittsburgh, Pennsylvania; § Ann & Robert H. Lurie Children’s Hospital of Chicago, Stanley Manne Children’s Research Institute, Research and Evaluation Center, Mary Ann & J. Milburn Smith Child Health Outcomes, Chicago, Illinois; || University of Pittsburgh Medical Center Children’s Hospital of Pittsburgh, Department of Critical Care Medicine, Pittsburgh, Pennsylvania

## Abstract

**Introduction:**

Timely identification of critically ill or injured children in prehospital and emergency settings remains a persistent challenge due to developmental variability, low case volumes in emergency medical services (EMS), and contextual limitations during field assessments. Existing frameworks to identify at-risk children often fail to capture the nuances of pediatric presentations, particularly in resource-limited or mass casualty settings. We aimed to explore prehospital and hospital-based clinician perspectives to inform a Delphi survey for the development of a consensus-driven definition of pediatric critical illness and injury.

**Methods:**

We conducted a qualitative study using one semi-structured interview and two focus groups with participants with expertise in pediatric prehospital and hospital acute care. Participants were presented with a list of tools commonly used to assess the severity of illness in children in the emergency department and hospital-based settings. Interviews were conducted virtually, transcribed, coded using an iterative process, and thematically analyzed. We used key themes to inform the structure and priorities for a future Delphi survey.

**Results:**

Six of the 12 invited participants took part in the study. Five major themes emerged: 1) prehospital indicators of critical illness (e.g., seizure, intravenous placement, cardiopulmonary resuscitation; 2) in-hospital markers of severity (e.g., air medical transport, intubation, diagnostic findings); 3) perceptions of existing triage tools (e.g., limited awareness or utility among paramedics); 4) differences in assessment approaches across roles and settings; and 5) specific triage challenges during mass casualty or disaster scenarios. Paramedics emphasized clinical actions as indicators of acuity, while physicians cited diagnostic findings and broader contextual indicators. Across roles, there was more agreement on the limitations of current triage and illness severity tools than on their utility.

**Conclusion:**

We gained insights into key gaps in current pediatric triage systems, including limited applicability of existing tools in prehospital settings, variability in comfort with pediatric interventions, and the lack of alignment between paramedic action-based indicators and physician reliance on diagnostic findings. Role-specific experiences influence how critical status is assessed and highlight the value of integrating multidisciplinary insight. These findings inform future work focused on the development of consensus-based outcome measures that align with decision-making across prehospital and hospital environments.

## INTRODUCTION

The accurate and timely identification of critically ill or injured pediatric patients in prehospital and emergency settings is a persistent challenge in emergency medicine.^1^–^3^ Pediatric presentations of acute conditions often differ significantly from those in adults, due to developmental variations,^2^ conditions encountered,^4^ and social complexities.^2^ Additionally, vital signs and other assessment information in children can fluctuate widely,^5^ even in minor emergencies. Prehospital emergency clinicians see far lower volumes of children compared to adults, inhibiting the rapid development of individual intuition for distinguishing critical illness in pediatric patients.^6^ Together, these factors introduce challenges in the triage process, transport decisions, and destination selection. Currently, there is a lack of standardization across settings in how children at risk for critical illness are assessed and managed.^7^ While emergency clinicians can rely on comprehensive assessments including clinical examinations, lab test results, and imaging data, prehospital clinicians often operate with limited time, tools, and contextual information.^1^–^3^

Research in adults has identified prehospital factors associated with high risk of subsequent in-hospital deterioration. One model, for example, combined hypotension and bradypnea with other variables obtained in the prehospital setting to identify adults with sepsis and those who will require mechanical ventilation or experience in-hospital mortality.^8^ Another study demonstrated that a model including heart and respiratory rate had superior performance compared to clinical gestalt for identifying patients with sepsis.^9^ Among children, the prediction of critical illness has been limited by the lack of a consensus definition of and criteria for clinically relevant outcomes, a fact that is particularly challenging given the low rates of in-hospital mortality among admitted children.^10^ Research to identify critical illness among children with out-of-hospital emergencies is scant, with one study demonstrating limitations of using expert-derived cutoffs for vital signs.^11^

More effective pediatric assessment tools and protocols are needed to ensure that care is prioritized correctly. Distinguishing between critical and non-urgent cases in routine prehospital encounters is essential for appropriately allocating resources, determining the safety of non-transport decisions, and guiding the destination for emergency care. Additionally, in disaster or surge scenarios, triage and destination decisions become even more consequential and bring additional challenges of constrained resources.^12^ In this qualitative study, we sought to capture perspectives from prehospital, emergency, and critical care clinicians on how pediatric critical illness is recognized across settings. By exploring role-specific decision-making processes and perceived gaps in current tools, we aimed to generate foundational themes to guide the development of a Delphi survey. Our ultimate goal in this body of work was to derive more effective, usable models for triaging children experiencing emergencies and disasters.

**Population Health Research Capsule**

What do we already know about this issue?

*Existing pediatric triage tools lack consensus and perform poorly in prehospital settings, making early identification of critical illness challenging*.

What was the research question?

*How do prehospital and hospital clinicians define and identify pediatric critical illness and injury?*

What was the major finding of the study?

*We identified five major themes and 12 subthemes describing clinician approaches to pediatric critical illness*.

How does this improve population health?

*Findings inform development of consensus tools to improve early recognition and triage of critically ill children across prehospital and hospital settings*.

## METHODS

### Study Design and Setting

We performed a qualitative study using semi-structured interviews and focus groups to explore how clinicians assess and identify critical illness in pediatric patients across prehospital and hospital settings. Performance of this study was approved by the Institutional Review Board at Ann & Robert H. Lurie Children’s Hospital of Chicago. We adhered to the Consolidated Criteria for Reporting Qualitative Research (COREQ) guideline.^13^

### Participants

Participants were recruited through purposive sampling to ensure representation from relevant clinical specialties (emergency medicine technician/paramedic, emergency physicians, critical care physicians) and institutions (children’s hospitals and general hospitals). All participants provided verbal consent and were compensated $35 for their time. Invitations were sent via email to 12 clinicians from the lead investigator’s professional and academic networks, with the intent of including perspectives from both children’s hospitals and general hospitals. Recruitment targeted clinicians with active pediatric emergency, prehospital, trauma, or critical care responsibilities, regardless of years of experience, and invitations were followed by one reminder email if no response was received. Six clinicians agreed to participate, representing a range of clinical environments, including academic and community-based practice settings.

We collected data through two small focus-group discussions and one individual interview. This combination allowed both in-depth exploration of individual experiences and collaborative discussion among clinicians. The sample size was determined pragmatically, based on availability and willingness of clinicians across the targeted disciplines to participate. While small, the sample provided the necessary perspectives from key professional roles relevant to pediatric prehospital and hospital emergency care to inform item development for a subsequent Delphi survey. Our intent was not to achieve statistical significance or population representativeness but to generate exploratory insights across disciplines.

### Interview Guide Development

We developed a semi-structured interview guide that allowed for iterative refinement based on emerging themes. In preliminary work, we reviewed relevant literature pertaining to prior research used to determine the presence of critical illness and/or injury among children in the emergency department,^14^–^19^ inpatient, and critical care settings.^20^,^21^ We developed a conceptual framework to identify factors associated with critical illness and/or injury and visualized relationships in a directed acyclic graph (Figure). The initial interview guide drew on elements from the graph and focused on topics such as the role of vital signs criteria, triage protocols, indicators of critical illness, and current assessment tools (Appendix). Pilot testing of interviews was done within the study group, with additional feedback obtained from pediatric emergency clinicians not involved with the study.

This directed acyclic graph illustrates hypothesized relationships between exposure (9-1-1 activation) and outcomes (eg, mortality, morbidities) in pediatric emergency care. The figure is intended to be read from the top left. The framework outlines key intermediary domains (including emergency medical system (EMS) response, ED arrival, assessment data, procedures/interventions/medications, diagnoses, hospital admission, and operating room use) that may influence or mediate the identification of critical illness or injury in children. Arrows represent proposed directional relationships between factors based on clinical reasoning and literature.

### Data Collection

All interviews were concurrently conducted by two individuals: a trained research professional experienced in qualitative methods with a BA in Biology (AH; female) and a pediatric emergency physician investigator who conducts research on predictive models for applications in clinical care (SR; male) using an online platform (Zoom Communications, San Jose, CA). The participants were familiar with the overall goal of the research program prior to the initiation of interviews. Participants were informed that the physician interviewer was conducting research to develop prediction models using EMS data to identify children with critical illness and/or injury.

Sessions lasted approximately 60 minutes. Initial questions pertained to how clinicians may assess critical illness and/or injury in the prehospital and in-hospital setting. Next, to provide a common foundation for discussion, participants were given a brief (five-minute) overview of the study aims and examples of existing illness severity tools. The interview guide focused on several key domains: clinicians’ current practices for distinguishing children who are sick vs not sick; how clinicians may interpret prehospital indicators of critical illness, including activation of protocols, measurements, interventions, and aspects of the patient care record most useful for triage; in-hospital indicators such as protocols, pathways, diagnoses, and outcomes signaling severity; perspectives on the utility and limitations of existing severity and triage tools; and anticipated changes in assessment and decision-making during disaster or mass casualty situations. After each session, members of the study team reviewed participant feedback and discussion flow, and minor adjustments were made to the phrasing and sequencing of questions to improve clarity and promote deeper engagement. Repeat interviews were not conducted.

### Analysis

All interviews were video- and audio-recorded with participant consent using Zoom and professionally transcribed, with transcripts verified for accuracy by the study team. After all interviews were complete, one transcript was chosen at random to be coded by two members of the study team (LB, AH) using an initial codebook developed from the interview guide. Coders independently coded the transcript. Transcripts were not returned to participants for comment. The team met to review and reach consensus on code application (addressing any disagreements that may have occurred in code application) and then revised the codebook. This process ensured consistency in coding and allowed for refinement of the thematic framework used in subsequent analysis. We assigned a primary and secondary coder to the remaining transcripts. The finalized codebook was applied across all transcripts to identify major themes and priorities. We used this process to generate consensus-based understanding of the clinical assessment of pediatric critical illness and to inform the development of decision support tools. Additional study team members who contributed to the interpretation of results were clinicians with experience in research and with varied clinical backgrounds (EMS, health services research, pediatric emergency medicine, and pediatric critical care).

## RESULTS

We conducted three semi-structured 1:1 and small-group interview sessions between February–April 2024. Participants included six clinicians: four physicians with backgrounds in pediatric emergency medicine, critical care, and trauma surgery; and two paramedics with research experience. Five of the participants were based in urban settings, and one in a suburban setting. Four of the clinicians worked in a children’s hospital, and two worked for local EMS systems. We identified several key themes related to the assessment of critical illness and injury in pediatric patients across prehospital and hospital settings through analysis of the transcripts.

These themes included the following: 1) prehospital indicators of critical illness; 2) in-hospital indicators of severity; 3) perceptions of existing clinical tools; 4) clinical assessment practices across settings and professional roles; and 5) considerations during disaster or mass casualty events. Within these themes, we identified 12 subthemes: procedural triggers of concern; mechanism of injury as a proxy; early physiological assessments; limitations of EMS impressions; transport mode as marker of acuity; monitoring and vital sign trends; diagnostic anchors of critical illness; knowledge and use gaps; practicality vs theory; dynamic nature of critical illness; institutional systems; triage and resource allocation; and contextual indicators of severity. A summary of these themes, with key quotes, is presented in the Table.

### Prehospital Indicators of Critical Illness

Participants identified several critical signs and interventions that, when observed in the field, signaled the potential presence of serious illness or injury in pediatric patients. Paramedics highlighted the presence of seizures and placed emphasis on the performance of procedures, including cardiopulmonary resuscitation, respiratory assistance, and placement of intravenous (IV) access as key indicators of critical illness in the out-of-hospital setting, reflecting the paramedics’ heightened concern. Notably, compared to adult patients, many prehospital clinicians reported limited comfort with pediatric IV insertion, which itself was recognized as a marker of critical illness. Unlike in adult EMS care, where IV access is often established routinely, the difficulty and hesitation surrounding pediatric IV placement reflect both the severity of the patient’s condition and the clinician’s recognition of a critical situation.

In contrast to prehospital clinicians who focused on procedures, physicians referenced a broader set of clinical and contextual indicators that may identify severity of illness or injury among children in the prehospital setting. These included abnormal vital signs (eg, heart rate, blood pressure, oxygen saturation), concerning mechanisms of injury, and the use of immobilization devices. A depressed Glasgow Coma Scale score, endotracheal intubation, and the need for vasopressors were also identified as strong markers of critical illness. While physicians generally valued prehospital observations, one participant expressed skepticism about using prehospital impressions alone to define critical status (see Table).

### In-Hospital Indicators of Severity

Although the care provided by prehospital clinicians typically concludes once a patient reaches the hospital, paramedics were asked to reflect on their thoughts about in-hospital factors that might indicate critical illness. One paramedic noted that in their geographic region, the use of air medical transport for a patient would strongly suggest critical illness. However, the participant acknowledged that this factor may not universally signify critical illness in other geographic settings, where the use of air transport might depend on different logistical or operational considerations.

Once the pediatric patient was in the hospital, physicians emphasized the importance of monitoring developmental appropriateness, changes in level of consciousness, and overall activity level. They considered clinical indicators, such as the need for a blood transfusion, presence of respiratory distress, or triggers for sepsis evaluation, strong signals of critical illness. Diagnostic imaging was also cited as essential for detecting severe conditions like spinal cord injuries or intracranial hemorrhage.

### Perceptions of Existing Clinical Tools

Participants were presented with a list of tools commonly used to assess the severity of illness in children presenting to the hospital via EMS, which included the following: Pediatric Risk of Hospital Admission,^22^ Revised Pediatric Emergency Assessment Tool,^15^ Emergency Severity Index (ESI),^14^ Pediatric Risk of Mortality (PRISM),^23^ the Pediatric Logistic Organ Dysfunction (PELOD) score,^21^ Injury Severity Score,^24^ need for emergent trauma intervention,^19^ need for emergent intervention within six hours, and standardized triage assessment tool.^18^ Physician respondents were generally familiar with three of these tools (ESI, PRISM, and PELOD), although they did not use them for decision-making.

When presented with existing tools for assessing severity of illness, participants expressed mixed familiarity and utility. Physicians were familiar with some of these tools but noted limitations in their practical use during acute triage. Paramedics reported minimal exposure to these tools and did not routinely use them in the field. Several participants emphasized that tools for use in the critical care setting are not available or useful in the early stages of care, particularly in prehospital contexts.

#### Clinical Assessment Across Settings and Professional Roles

Both physicians and paramedics discussed how children are currently assessed in their professional settings to determine whether they are critically ill or injured. Across professional roles, clinicians reported similar foundational assessment steps: obtaining vital signs; evaluating airway, breathing, and circulation; and assessing overall clinical appearance. However, differences emerged in how these assessments were contextualized. Paramedics often relied on caregiver reports at the scene and followed Pediatric Advanced Life Support guidelines. In contrast, physicians incorporated additional data such as lab values, imaging, and tiered triage systems. Changes in assessment over the period of transport were also noted.

### Disaster and Mass Casualty Contexts

Participants noted that triage and care practices shift significantly in disaster or mass casualty situations. Clinicians reported that decisions must often be made in resource-constrained environments, with adaptations such as providing care in non-traditional spaces and prioritizing patients based on survivability. Physicians emphasized that environmental context (eg, presence of other fatalities, chemical exposures) plays a larger role in assessing severity in such circumstances.

## DISCUSSION

We collected qualitative data through discussions with clinicians to evaluate factors that may be indicative of clinically important illness and/or injury in children to inform a Delphi process for criteria that will ultimately be used in models for risk stratification. A wide array of approaches and prehospital factors that could potentially signal critical illness were reported, with clinicians generally noting limitations with existing systems used to risk stratify children with acute illness in the hospital. These findings may be used in the development of structured processes to determine outcomes for critical illness and/or injury for children in the prehospital setting.

The variability in assessment methods between paramedics and physicians underscores the need for a structured framework to ensure consistency in identifying critically ill children. Prior research has identified challenges in the prehospital assessment of children, a finding that highlights opportunities to improve the prehospital assessment.^1^ We noted variable experiences that aligned with the different professional positions of the participants (ie, prehospital clinicians vs in-hospital physicians) and settings (ie, field vs emergency. department). The identified differences in assessment processes reflect the real-world constraints, decision points, and informational inputs available to each group. These distinctions are important to the development of Delphi items intended to create consensus-based outcome criteria for critical illness and/or injury for children transported to the hospital by EMS.

Our findings have broader implications for patient care and system design. By characterizing role-specific challenges and limitations of existing tools, this qualitative work provided the foundation for our subsequent Delphi process, which was intended to achieve consensus on data elements to include in hospital-based outcome measures for pediatric critical illness and injury among children who receive prehospital care. Establishing a structured definition through this stepwise approach can inform training, guide triage and transport decisions, and reduce variability in care, ultimately supporting safer and more consistent management of children across EMS systems. For example, paramedics were focused on procedural factors as being indicative of a higher level of concern (eg, peripheral IV line placement), suggesting an emphasis on clinical action by treating personnel. Notably, this approach does not capture the underlying reasoning for why an intervention was performed. For example, vascular access may be obtained as a precautionary measure by EMS clinicians who want to ensure access is available en route rather than because vascular access is necessary for treatment of critical status. Our data did not disentangle these contextual motivations or examine procedural interventions in relation to their triggers. In comparison, physician criteria were broader and incorporated information such as mechanism of injury, vital signs, diagnostic testing, and the performance of in-hospital testing.

We found that perceptions of different structured rubrics also aligned with clinical background; all were identified to have limitations when considered as measures of critical illness and/or injury among children transported to the hospital by EMS. Paramedics reported relying on Pediatric Advanced Life Support algorithms and the use of prehospital protocols (particularly for cardiac arrest) to determine for patient acuity. One qualitative study of 17 paramedics identified several factors that make pediatric calls particularly difficult, specifically citing clinical complexities associated with pediatric patients and emphasizing the unique difficulties of determining acuity in the prehospital setting.^2^ Given the limited role of vital signs in predicting the need for key interventions (including potentially life-saving interventions)^25^,^26^ and in-hospital outcomes,^11^,^27^ these findings underscore the importance of standardized clinical decision support tools and structured assessment frameworks in prehospital care. They also underscore the need to better define and validate field indicators of critical illness beyond vital signs alone, particularly in pediatric populations.

The results of our thematic analysis provide a foundation for item generation and prioritization in the early stages of tool or score development, especially for use in the prehospital setting. These include the incorporation of diagnoses, injury mechanisms, medications, interventions, and dispositions, which may inform the development of candidate criteria in future work. Our findings emphasize the value of incorporating multidisciplinary insights and perspectives on certain interventions (such as prehospital venous access in children) that may be useful as proxy measures for illness or injury severity. Further, the findings from this study provide several avenues to develop comprehensive frameworks to stratify children in the prehospital setting based on level of acuity. Participants identified limitations in current triage and severity tools, emphasizing the need for simple, real-time decision support adapted for pediatric care.

We undertook this qualitative effort to inform the design of a Delphi survey focused on the development of consensus-based outcome measures to define critical illness and injury. Importantly, the small sample size and participation rates carry implications beyond simple limitations. Only half of those invited participated, largely due to scheduling conflicts. Our recruitment efforts were focused on clinicians with greater exposure to the care of children with critical illness and injury, primarily those based at children’s hospitals and in metropolitan areas. This approach allowed us to draw on the experience of clinicians who care for a higher volume of critically ill or injured children. In doing so, however, we did not recruit from community or rural settings and, thus, excluded the perspectives of clinicians practicing in those environments. While ensuring cross-disciplinary representation (EMS, emergency and critical care), this likely over-represents academic viewpoints. Future work could use these results and drive toward thematic saturation by broadening recruitment strategies to capture additional experiences and perspectives from community and rural providers. Perspectives from first responders in rural areas with longer transport times may have yielded unique perspectives on decision-making in those contexts.

## LIMITATIONS

Our findings are subject to limitations. While we conducted both individual and small-group interviews, the small sample size limits the generalizability of findings, although they remain valuable for informing the development of Delphi survey items. Our timeline was compressed to achieve the aim of developing the items within a short grant period. Participants were largely from academic or pediatric institutions in urban settings, which may be less reflective of experiences in community or rural settings and other parts of the US. Additionally, the views expressed may disproportionately reflect the experiences of physicians and paramedics with research backgrounds, potentially overlooking insights from clinicians with different roles or levels of research experience.

The fact that participants were aware of the interviewers’ professional credentials and research focus may have influenced what they felt comfortable disclosing, particularly regarding uncertainties in pediatric care. We included a presentation within the session to ensure clarity in terminology, and we acknowledge this may have influenced participant responses. While we included paramedic respondents, just two participated. Many of the existing triage and severity tools that were presented during the sessions were unfamiliar or underused by prehospital personnel, which may have limited meaningful evaluation of the applicability of these tools in early field care. Although recurring concepts emerged across interviews, we cannot be certain that thematic saturation was reached. Additional participants from different geographic areas could potentially have contributed new perspectives. Thus, our findings should be viewed as exploratory and hypothesis-generating, serving as a foundation for future consensus work. Despite these limitations, the study offers valuable insights into current practices and considerations that can inform the development of more effective, consensus-driven criteria for identifying pediatric critical illness and injury.

## CONCLUSION

We identified key factors used by clinicians to assess critical illness in pediatric patients across prehospital and hospital settings, revealing both common practices and role-specific differences. These insights support the need for the development of a structured, consensus-driven outcome measures that reflects real-world clinical judgment and can enhance triage, risk stratification, and decision-making in emergency care for children.

## Supplementary Information



## Figures and Tables

**Figure:**
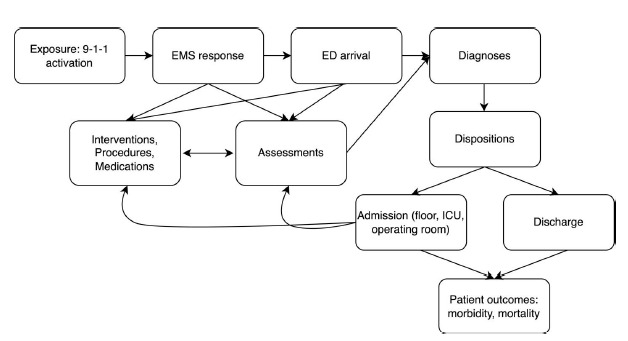
Conceptual framework of factors relevant to the identification of critical illness or injury among children transported to the hospital by emergency medical services. *EMS*, emergency medical services; *ED*, emergency department; *ICU*, intensive care unit.

**Table:** Summary of key themes regarding indicators of pediatric critical illness with representative participant quotes obtained through semi-structured interviews of clinicians.

Theme	Sub-themes	Participant quote
Prehospital indicators of critical illness	Procedural triggers of concern	“I think a majority of paramedics are only mildly comfortable starting IVs, especially if you think of very small children. So if I’m reading a chart of a kid, say under 5, and I see intra(venous) access that immediately makes me think, okay, they recognize something, whether it be the level consciousness, blood pressure, whatever where they thought immediate intervention was necessary. A lot of paramedics do that sort of balance where they think like, I’m not comfortable with this. I don’t really wanna do it so am I sure that I need to? So if then they perform the action, it speaks to [the idea that they] see something that is critically wrong.” Participant 1d (Paramedic)
	Mechanism of injury as a proxy	“I think is a proxy for an assessment by the prehospital provider of the significance of a mechanism of injury like in what we’re talking about blunt trauma. Specifically, whether it’s a fall or a collision of some type of placement of a long spine board with a mobilization or rigid cervical collar. Both make me think that the prehospital provider was also worried about a potential significant mechanism of injury, and then therefore potentially a resultant critical injury.” Participant 1b (Physician)
	Early physiological assessments	“Appropriate Glasgow Coma Scale assessments for prehospital providers could be very helpful. Because, again, that is a quick initial assessment that can give some information about the child status globally.” Participant 1b (Physician)
	Limitations of EMS impressions	“I wonder if we’re all dancing around the subject. Because I think that, short of the patient (who) required emergency intubation in the field, I don’t want to speak for others, but I’m reflecting my own biases that EMS’s impression should not determine critical illness.” Participant 1a (Physician)
In-hospital indicators of severity	Transport mode as marker of acuity	“One of the biggest things that we see is just interfacility transport from the local hospital to the children center and this occurs often by ground…. So the children that we do fly are often what I would consider to be critically ill or injured, severe sepsis, intubated, receiving blood products, multi system, trauma, things like that, status epilepticus... But I think, at least from my experience, just the mere presence of air medical transport from a local hospital to a children’s center is a pretty reliable indicator.” Participant 1d (Paramedic)
	Monitoring and vital sign trends; diagnostic anchors of critical illness	“And so, as we look at regular routine monitoring, what kind of things are we looking at for those flags and then paying attention to the fever as well bring that fever down, and that bronchiolitis no longer breathing 80 and so I think some of those things, I think diagnoses are hard if you’re looking prospectively. And then, of course, trauma so those kind of diagnoses with critical fractures, life and limb, threatening and spinal cord injuries, too.” Participant 2a (Physician)
Perceptions of clinical gools	Knowledge and use gaps	“I knew of the injury severity score when I was practicing with the clinician, but we didn’t use any of these other types of scales and from an EMS educational standard standpoint, it’s not a part of the education… I don’t know that a lot of prehospital clinicians are going to be considering some of these things.” Participant 2d (Paramedic)
	Practicality versus theory	“. . . So I think the thing that strikes me looking at these is that none of them will be available prehospital, and most of them won’t even be known within the first minutes to hours of the in-hospital assessment. Many of them not until days later.” Participant 1b (Physician)
Clinical assessment across settings and professional roles	Dynamic nature of critical illness	“One distinction that’s worth making or teasing out is whether they’re critically ill at their initial presentation versus they are critically ill after they respond to initial resuscitation... if they recover within an hour and they go to the general ward they’re less ill than someone who has escalating interventions and pressures, etc, over the next 48 hours. And it what cut off does that matter?” Participant 1a (Physician)
	Institutional systems	“We have a tier system, which is a system for the emergency department physician to identify children who they believe have significant illness, that may require critical care and can essentially say you are, you know, tier 2 would mean based on our intervention we’ve done, this (patient) needs critical care moving forward, and we need to expedite having it.” Participant 3a (Physician)
Disaster and mass casualty. contexts	Triage and resource allocation	“I think, as with anything, the triage process is what changes, and when you have to start considering survivability and resource allocation to do the greatest good for the greatest number is the only time when that calculus really changes, and that can be something that’s very difficult… So you might not be able to do things in your big fancy trauma bay with a dozen providers all around the patient… You may have to utilize spaces in creative ways, and delegate and sort of do field promotions for trainees with less oversight and less providers per patient.” Participant 1b (Physician)
	Contextual indicators of severity	“I think, as with any traumatic injury that affects more than one person, you can glean severity from whether there were other fatalities on scene… whether it’s a mass casualty disaster or otherwise unexpected mechanistic- or exposure-related information is probably more useful in that setting, especially if it’s something otherwise intentional that could involve chemical, biologic, or nuclear exposure radiation is what I mean by that.” Participant 1b (Physician)

*EMS*, emergency medical services; *ICU*, intensive care unit; *IV*, intravenous line.
