# Supplementary material for: Prehospital and Emergency Care Perspectives to Define Pediatric Critical Illness and Injury
Source: West J Emerg Med. 2026 Jan 9;27(1):121–9. doi: 10.5811/westjem.48526 (PMC12815507; doi:10.5811/westjem.48526)
Supplement: Supplementary file 1 [file wjem-27-121-s001.docx]

**Prehospital and In Hospital Outcomes**

**Focus Group Guide**

Welcome and thank you for taking the time to participate in this discussion today. My name is [moderator] and I am a [job title] at Lurie Children’s Hospital. We’ve asked you to participate in this conversation today because you care for children in the pediatric setting. We are working to develop a prediction model for children to identify critical illness and clinical decompensation based on vital signs and other clinical data. We’d like to hear your thoughts and knowledge on this topic.

This discussion will be recorded, so we have an accurate transcript, however we will not use anyone’s name or identifying information in any reports, changes, upgrades, etc. You have the right to pass on any question that you don’t feel comfortable answering.

This discussion is meant to be a learning experience for all of us. We want to hear as many perspectives as possible. Therefore, we want to be clear that there are no right or wrong answers, only perspectives and experiences, all valid in their own right. Additionally, we realize disagreements may come up, and that is okay – we just ask that everyone treat each other with respect.

Does anyone have any questions before we get started?

**Introduction**

**Introduce study team members and allow participants to introduce themselves.*

We’d like to start by discussing the overall purpose of our study.

1. In your professional settings, how are children currently assessed to determine if they are sick or not sick?
2. Which prehospital protocols, measurements, interventions or signs and symptoms signal the presence of critical illness or injury in children to you?
   1. Which other aspects of the prehospital care record do you believe are most useful in the triage of children with prehospital emergencies and disasters?
3. Which in-hospital protocols or pathways, measurements, interventions, diagnoses, or clinical outcomes signal the presence of critical illness or injury in children to you?
4. Now we’re going to look at some existing measures that are used to determine critical illness for children. What are the pros and cons of these?
5. How would your approach change during disasters or mass casualty incidents?

Thank you for all the input you’ve shared today. As a reminder, you will receive a $35 gift card for your time and participation in today’s discussion. Are there any questions before we end today?
